# Dynamic pressure–volume loop analysis by simultaneous real-time cardiovascular magnetic resonance and left heart catheterization

**DOI:** 10.1186/s12968-023-00913-4

**Published:** 2023-01-16

**Authors:** Felicia Seemann, Christopher G. Bruce, Jaffar M. Khan, Rajiv Ramasawmy, Amanda G. Potersnak, Daniel A. Herzka, John W. Kakareka, Andrea E. Jaimes, William H. Schenke, Kendall J. O’Brien, Robert J. Lederman, Adrienne E. Campbell-Washburn

**Affiliations:** 1 Cardiovascular Branch, Division of Intramural Research, National Heart, Lung, and Blood, Institute, National Institutes of Health, 10 Center Drive, Building 10 Rm B1D47, Bethesda, MD 20892 USA; 2 Instrumentation Development and Engineering Application Solutions, Division of Intramural Research, National Institute of Biomedical Imaging and Bioengineering, National Institutes of Health, Bethesda, MD 20892 USA

**Keywords:** Pressure–volume loops, Myocardial contractility, Myocardial compliance, CMR-guided catheterization, Real-time CMR

## Abstract

**Background:**

Left ventricular (LV) contractility and compliance are derived from pressure–volume (PV) loops during dynamic preload reduction, but reliable simultaneous measurements of pressure and volume are challenging with current technologies. We have developed a method to quantify contractility and compliance from PV loops during a dynamic preload reduction using simultaneous measurements of volume from real-time cardiovascular magnetic resonance (CMR) and invasive LV pressures with CMR-specific signal conditioning.

**Methods:**

Dynamic PV loops were derived in 16 swine (n = 7 naïve, n = 6 with aortic banding to increase afterload, n = 3 with ischemic cardiomyopathy) while occluding the inferior vena cava (IVC). Occlusion was performed simultaneously with the acquisition of dynamic LV volume from long-axis real-time CMR at 0.55 T, and recordings of invasive LV and aortic pressures, electrocardiogram, and CMR gradient waveforms. PV loops were derived by synchronizing pressure and volume measurements. Linear regression of end-systolic- and end-diastolic- pressure–volume relationships enabled calculation of contractility. PV loops measurements in the CMR environment were compared to conductance PV loop catheter measurements in 5 animals. Long-axis 2D LV volumes were validated with short-axis-stack images.

**Results:**

Simultaneous PV acquisition during IVC-occlusion was feasible. The cardiomyopathy model measured lower contractility (0.2 ± 0.1 mmHg/ml vs 0.6 ± 0.2 mmHg/ml) and increased compliance (12.0 ± 2.1 ml/mmHg vs 4.9 ± 1.1 ml/mmHg) compared to naïve animals. The pressure gradient across the aortic band was not clinically significant (10 ± 6 mmHg). Correspondingly, no differences were found between the naïve and banded pigs. Long-axis and short-axis LV volumes agreed well (difference 8.2 ± 14.5 ml at end-diastole, -2.8 ± 6.5 ml at end-systole). Agreement in contractility and compliance derived from conductance PV loop catheters and in the CMR environment was modest (intraclass correlation coefficient 0.56 and 0.44, respectively).

**Conclusions:**

Dynamic PV loops during a real-time CMR-guided preload reduction can be used to derive quantitative metrics of contractility and compliance, and provided more reliable volumetric measurements than conductance PV loop catheters.

**Supplementary Information:**

The online version contains supplementary material available at 10.1186/s12968-023-00913-4.

## Introduction

Left ventricular (LV) contractility and compliance are valuable parameters to evaluate cardiac function and impact of an intervention [1–3]. Contractility and compliance can respectively be quantified from the slopes of the end-systolic and end-diastolic pressure–volume (PV) relationship (ESPVR, EDPVR) [2, 4, 5]. The ESPVR and EDPVR are derived from dynamic LV PV loops during a preload alteration challenge, traditionally derived with a conductance PV loop catheter while occluding the inferior vena cava (IVC) [6]. Conductance PV loop catheters measurements are reproducible but prone to unreliable volume measurements, despite extensive calibration typically requiring multiple hypertonic saline injections and a secondary independent stroke volume measurement [7–9].

Methods to record PV loops by combining volumetric measurements using echocardiography or cardiovascular magnetic resonance (CMR) and pressure measurements using a catheter have previously been proposed [10–15]. The most reliable method to determine intracardiac volumes is CMR [16], but lengthy acquisitions and low temporal resolution hampers dynamic imaging for PV loop recordings. Additionally, CMR generates interference on physiological signal recordings; specifically gradient- and radiofrequency (RF) induced interference on electrocardiogram (ECG) recordings, and RF-induced interference on invasive blood pressure. A technique to determine contractile function from dynamic PV loops using real-time CMR in a single short-axis slice has previously been proposed [13], but relied on a volumetric calibration, and did not filter CMR-induced noise from the pressure signals.

In this study, we propose a method to quantify contractility and compliance from dynamic PV loop measurements using real-time CMR during IVC occlusion. The method combines simultaneous measurements of invasive LV blood pressure with CMR-specific signal conditioning [17], and volume derived from a long-axis, real-time, CMR without the need for calibration. The method was tested in healthy swine and in two porcine disease models and compared with conductance catheter measurements.

## Methods

Dynamic PV loop experiments were performed in a combined X-Ray/0.55 T CMR cardiovascular catheterization laboratory. Dynamic PV loops were obtained in CMR, at end-expiratory breath-hold, during an abrupt IVC occlusion by balloon inflation. To capture ventricular preload-induced dynamics before significant sympathetic activation, we simultaneously acquired real-time CMR images to derive LV volume and LV pressure catheterization before and during IVC occlusion.

### Animal models

Animal experiments were approved by the local Institutional Animal Care and Use Committee and conducted per contemporary guidelines from the National Institutes of Health. We prospectively performed 16 dynamic PV loop experiments in female juvenile Yorkshire swine. Three porcine models were studied: naïve (n = 7, 50 ± 6 kg), chronic afterload increase by aortic banding (n = 6, 46 ± 7 kg), and ischemic cardiomyopathy (n = 3, 83 ± 9 kg). Induction of a chronic afterload increase was created by inserting a transcatheter external aortic band [18]. In short, pericardial access was obtained through percutaneous subxiphoid access. A guidewire loop was formed around the ascending aorta within the pericardial sac using deflectable catheters, which was subsequently exchanged for a suture band secured in position with a Rumel-style tourniquet and subxiphoid pocket [19]. Ischemic cardiomyopathy was induced as a model of impaired systolic function induced by multi-vessel transcoronary ethanol chemoablation of the mid left anterior descending coronary artery and an obtuse marginal branch. The chronic afterload increase model pigs were examined 30 days after aortic band insertion, and cardiomyopathy pigs were examined 154 (142–161) days after chemoablation.

Animals were maintained under general anesthesia with mechanical ventilation and isoflurane inhalation. Percutaneous femoral arterial and venous access was obtained. Under X-ray, a fluid-filled dual-lumen catheter (*Langston*, Teleflex, Morrisville, North Carolina, USA) was placed in the LV to allow simultaneous recording of LV and aortic pressures, and a non-metallic polymer based 24 mm air filled balloon catheter (*Atlas,* Bard, New Providence, New Jersey, USA) was positioned within the right atrium (RA). Animals were transferred to the adjacent CMR scanner for further real-time CMR-guided catheterization [20, 21]. Preload reduction was obtained under CMR guidance by fully inflating and withdrawing the balloon to occlude the IVC-RA junction. Intravenous heparin to achieve an activated clotting time > 250 s and amiodarone were administered.

### CMR PV loop measurement

Imaging was performed using a contemporary 0.55 T CMR system (prototype MAGNETOM Aera, Siemens Healthineers, Erlangen, Germany) [22]. Real-time long-axis 4-chamber cardiac CMR cine images were acquired during end-expiratory breath-hold using a Cartesian balanced steady-state-free-precession sequence (TE/TR/θ = 1.4 ms/3.3 ms/80°, 2.3 × 2.3 × 8 mm reconstructed resolution, 360 × 270 mm FOV, acceleration rate 3, 76 ms temporal resolution, 326 timeframes). The IVC-occlusion was performed ~ 5 s after the start of imaging, and deflated after an additional 15–20 s.

A custom, open source, hemodynamic recording system (PRiME, Physiological Recording in the CMR Environment) was used to record LV pressures [17]. PRiME allows synchronized recording of a six lead ECG, two invasive blood pressures, and CMR gradient waveforms at a sampling frequency of 1 kHz, and supports cardiac triggering from either the ECG or pressure signal. The recorded gradient activity provided a temporal fiducial to ensure that only physiological signals recorded simultaneously with the real-time CMR acquisition were sampled for the PV signal pairing.

### Signal processing

**Fig. 1:**
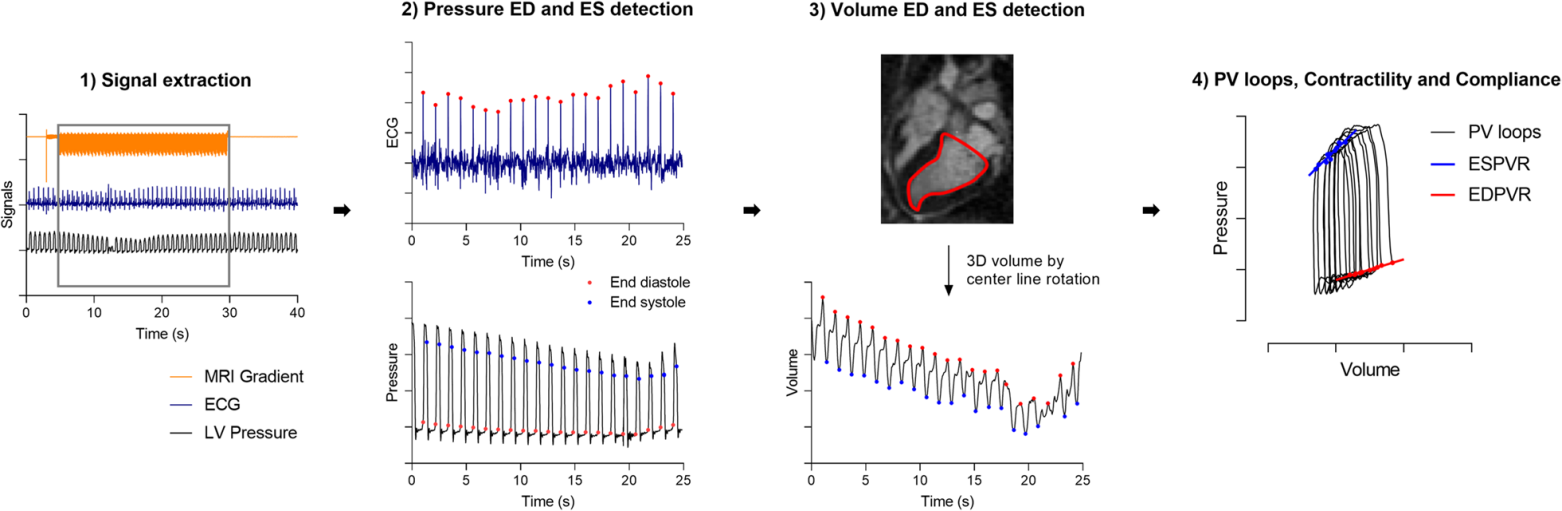
Dynamic pressure–volume (PV) loops were acquired during preload alteration challenge by inferior vena cava (IVC) occlusion. **1** Extraction of the portion of the pressure and electrocardiogram (ECG) signals recorded simultaneously to imaging were detected from the cardiovascular magnetic resonance (CMR) gradient activity (grey rectangle). **2** In the first ten heartbeats post balloon inflation, end-diastole (red) was detected from the R peaks in the ECG and end-systole (blue) as the onset isovolumic relaxation in the pressure signal. **3** 3D left ventricular (LV) volume was derived from long-axis segmentations. End-diastole and end-systole were detected as the maximum and minimum volumes, respectively. **4** PV loops were derived by pairing pressure and volume. Contractility and compliance were derived from the slopes of the end-systolic pressure–volume relationship (ESPVR) and end-diastolic pressure–volume relationship (EDPVR) linear fits, respectively

An automated signal processing pipeline that derives PV loops, contractility and compliance in six steps was implemented (Fig. 1):

1. Signal extraction: To synchronize pressure and volume, the portion of the recorded pressure and ECG signals acquired simultaneously to image acquisition was detected from the recorded CMR gradient waveforms, followed by a fourfold down-sampling of the signal from 1 kHz to 250 Hz.
2. Pressure: End-diastole was detected from R-peak in ECG signal, and end-systole from the onset isovolumic relaxation in the LV pressure. End-diastolic detections were then populated to the simultaneously sampled LV pressure signal.
3. Volume: Time-resolved 2D LV segmentation was performed semi-automatically on the 4-chamber images, with manual corrections as necessary (*suiteHEART*, NeoSoft, LLC, Pewaukee, Wisconsin, USA). From these, a 3D LV volume was derived using center line rotation.
  End-diastole and end-systole were detected as the maximum and minimum volume recorded in each heartbeat, followed by a signal up-sampling from the 73 ms temporal resolution to match the 250 Hz (4 ms temporal resolution) of the down-sampled pressure signal.
4. PV loops: PV loops were derived by pairing and aligning the pressure and volume signals based on the end-diastolic and end-systolic detections. We used only the first 10 heartbeats after balloon inflation, to avoid autonomic tone impact on inotropy and loading conditions.
5. Contractility: Contractility was calculated as the slope of the ESPVR, which was derived by performing a linear fit of the end-systolic detections in the PV loops.
6. Compliance: EDPVR was derived by fitting the end-diastolic detections in the PV loops to a linear function, and compliance was calculated as the inverse of the slope of the EDPVR at end-diastole.

The pipeline was implemented in MATLAB (Mathworks, Natick, Massachusetts, USA), allows manual corrections of end-diastolic and end-systolic detections, and is available as open source (https://github.com/NHLBI-MR/dynamic_pv_loops).

### Conductance catheter protocol

The PV loops measured in the CMR environment were compared to same day conductance PV loop catheter measurements in the X-Ray lab in a subset of 5 animals (n = 2 naïve and n = 3 cardiomyopathy). Dynamic PV loop catheter measurements were performed during IVC occlusion under X-ray fluoroscopy guidance, breath-held at end-expiration, using a 7F conductance PV loop catheter and PV loop system (*signa-M*, Leycom, Hengelo, The Netherlands). The PV loop catheter was calibrated in two steps according to the manufacturer manual instructions. First, the myocardial parallel conductance was determined by injecting hypertonic saline boluses, enabling the catheter to provide relative volumetric measurements. Second, absolute end-diastolic volume (EDV), end-systolic volume (ESV), and ejection fraction were calibrated using same-day volumetric ECG-gated short-axis-stack measurement from CMR. This calibration procedure is performed once in the beginning of a PV loop experiment at baseline, meaning that the calibration is not performed beat-to-beat. Dynamic PV loops were measured during IVC occlusion at a sampling frequency of 250 Hz. End-diastole and end-systole were detected as the maximum and minimum volume recorded in each heartbeat, respectively. Contractility and compliance were derived as described above, from the slopes of the linear fits of the end-systolic and end-diastolic detections in the 10 first recorded heartbeats after IVC occlusion.

### Volumetric validation

To validate volumes calculated by real-time long-axis CMR, breath-held retrospectively cardiac gated balanced steady state free precession short-axis-stack cine images (TE/TR/θ = 1.79 ms/4.4 ms/78°, 0.7 × 0.7 × 8 mm reconstructed resolution, 360 × 270 mm FOV, 16 slices covering the left ventricle (LV), bandwidth 305 Hz/Pixel, 34 ms temporal resolution, 30 timeframes) were acquired in all animals, without balloon inflation. Short-axis LV segmentations at end-diastole and end-systole were performed using the freely available software Segment (v3.1 R8154, Medviso, Lund, Sweden) [23]. The same segmentations were used for the conductance catheter calibrations. EDV and ESV LV volumes measured with real-time long-axis CMR and with a conductance catheter, from the first recorded heartbeat prior to balloon inflation, were compared to short-axis-stack volumes.

### Statistical analysis

Statistical analysis was performed using GraphPad Prism 9 (GraphPad Software, Inc, La Jolla, CA, USA). Continuous variables were reported as mean ± standard deviation (SD). Contractility and compliance between naïve and diseased animal models were compared using one-way ANOVA. Agreement between measured long-axis and short-axis derived LV volume were described with Bland–Altman analysis and intraclass correlation coefficient (ICC), where levels of agreement was defined as poor (0.00–0.30), weak (0.31–0.50), moderate (0.51–0.70), strong (0.71–0.90), and excellent (0.91–1.00) [24]. Pearson R were reported and statistical significance was considered for p < 0.05. Differences between parameters derived from PV loops measured in the CMR environment and with a conductance PV loop catheter was assessed with a paired t-test.

## Results

**Fig. 2:**
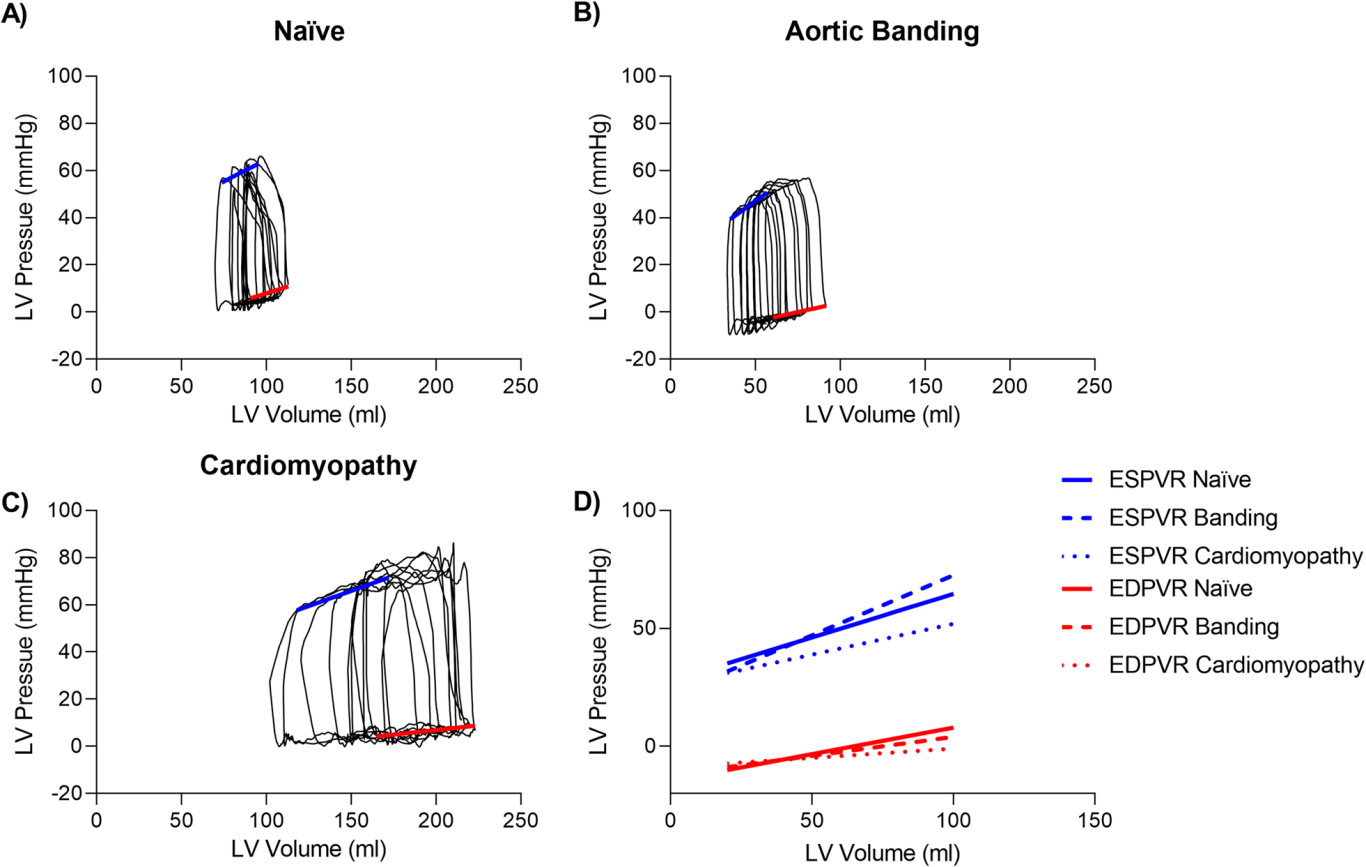
Examples of ten consecutive derived PV loops (black) with corresponding ESPVR (blue) and EDPVR (red) lines in **A** a naïve pig, **B** a pig 30 days after aortic banding, and **C** pig 30 days after induction of ischemic cardiomyopathy. **D** Combined ESPVR and EDPVR from naïve, banded, and cardiomyopathy pigs. The modest pressure gradient over the aortic banding resulted in similar results compared to naïve pigs

Volumetric short-axis CMR measurements and LV pressures are reported in Table 1 for the three animal models. Invasive LV pressure and real-time long-axis imaging during IVC-occlusion was feasible in the CMR scanner, and PV loops were derived in all experiments. An example cine image and corresponding LV volumes acquired during IVC occlusion is shown in Additional file 1: Video S1. The same number of heartbeats were independently detected in the pressure and volume signals in all experiments. Examples of dynamic PV loops and corresponding ESPVR and EDPVR are shown in Fig. 2. Over the course of the 10 included heartbeats, IVC occlusion induced an 18 ± 5% reduction in peak systolic LV pressure, a 30 ± 17% reduction in end-systolic volume, and a 0.8 ± 4.8% increase in heart rate.

**Table 1:** Characteristics of animal models measured from short-axis CMR images and fluid-filled catheters

	Naïve (n = 7)	Aortic banding (n = 6)	Cardiomyopathy (n = 3)
Weight (kg)	50 ± 6	46 ± 7	83 ± 9****
Heart rate (bpm)	77 ± 16	82 ± 18	68 ± 5 ns
EDV (ml)	111 ± 15	109 ± 20	277 ± 36****
ESV (ml)	70 ± 20	68 ± 23	204 ± 28****
SV (ml)	41 ± 14	41 ± 5	73 ± 16**
EF (%)	39 ± 12	36 ± 10	27 ± 5
CO (l/min)	3.3 ± 1.7	3.4 ± 0.9	5.0 ± 1.4
LV EDP (mmHg)	9.2 ± 4.4	6.1 ± 3.3	12.3 ± 1.6
Peak LV pressure (mmHg)	74.9 ± 8.0	74.6 ± 11.0	84.3 ± 2.7
Aortic SBP (mmHg)	69.1 ± 5.9	64.4 ± 10.4	77.5 ± 2.0
Aortic DBP (mmHg)	49.0 ± 3.8	47.0 ± 8.6	56.0 ± 10.4

Parameters HR, EDV, ESV, SV, and CO are reported from the short-axis cine images. Pressures are reported from the first recorded heartbeat prior to inferior vena cava occlusion in the dynamic pressure–volume loop experiment. Continuous variables are reported as mean ± SD. *EDV* end-diastolic volume, *ESV* end-systolic volume, *SV* stroke volume, *EF* ejection fraction, *CO* cardiac output, *LV* left ventricular, *EDP* end-diastolic pressure, *SBP* systolic blood pressure, *DBP* diastolic blood pressure. Differences from naïve animals by one-way ANOVA: *p < 0.05; **p < 0.01, ****p < 0.001

### Validation of left ventricular volume measurement

**Fig. 3:**
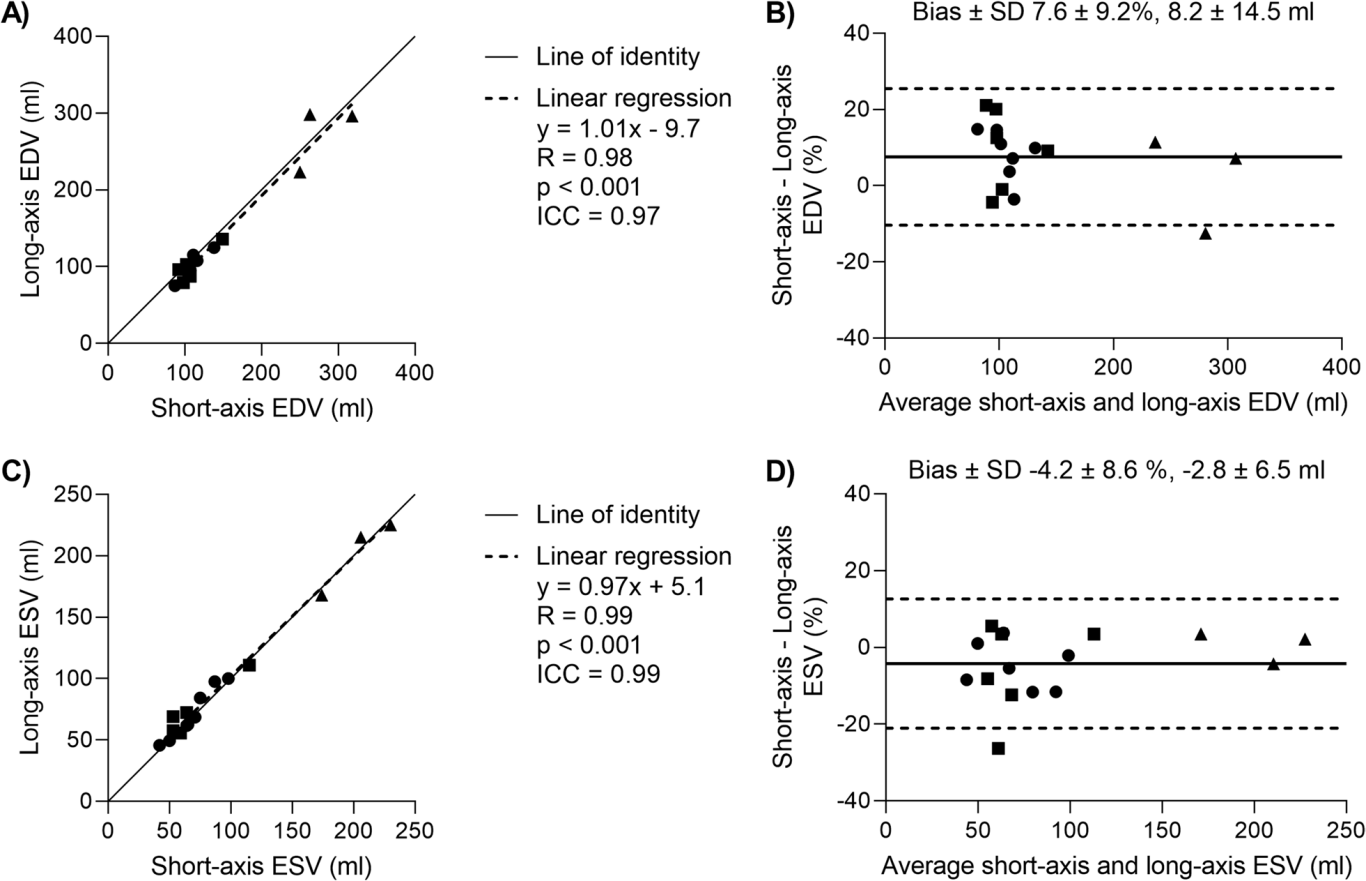
Comparison of short-axis and long-axis measurements of LV end-diastolic and end-systolic volumes (EDV, ESV). Naïve animals are shown as circles, aortic banded animals as squares, and ischemic cardiomyopathy animal models as triangles. **A** Scatter plot of EDV. **B** Bland–Altman plot of EDV. Bias and limits of agreement are shown as solid and dashed lines, respectively. **C** Scatter plot of ESV. **D** Bland–Altman plot of EDV. ICC, intraclass correlation coefficient; SD, standard deviation

**Fig. 4:**
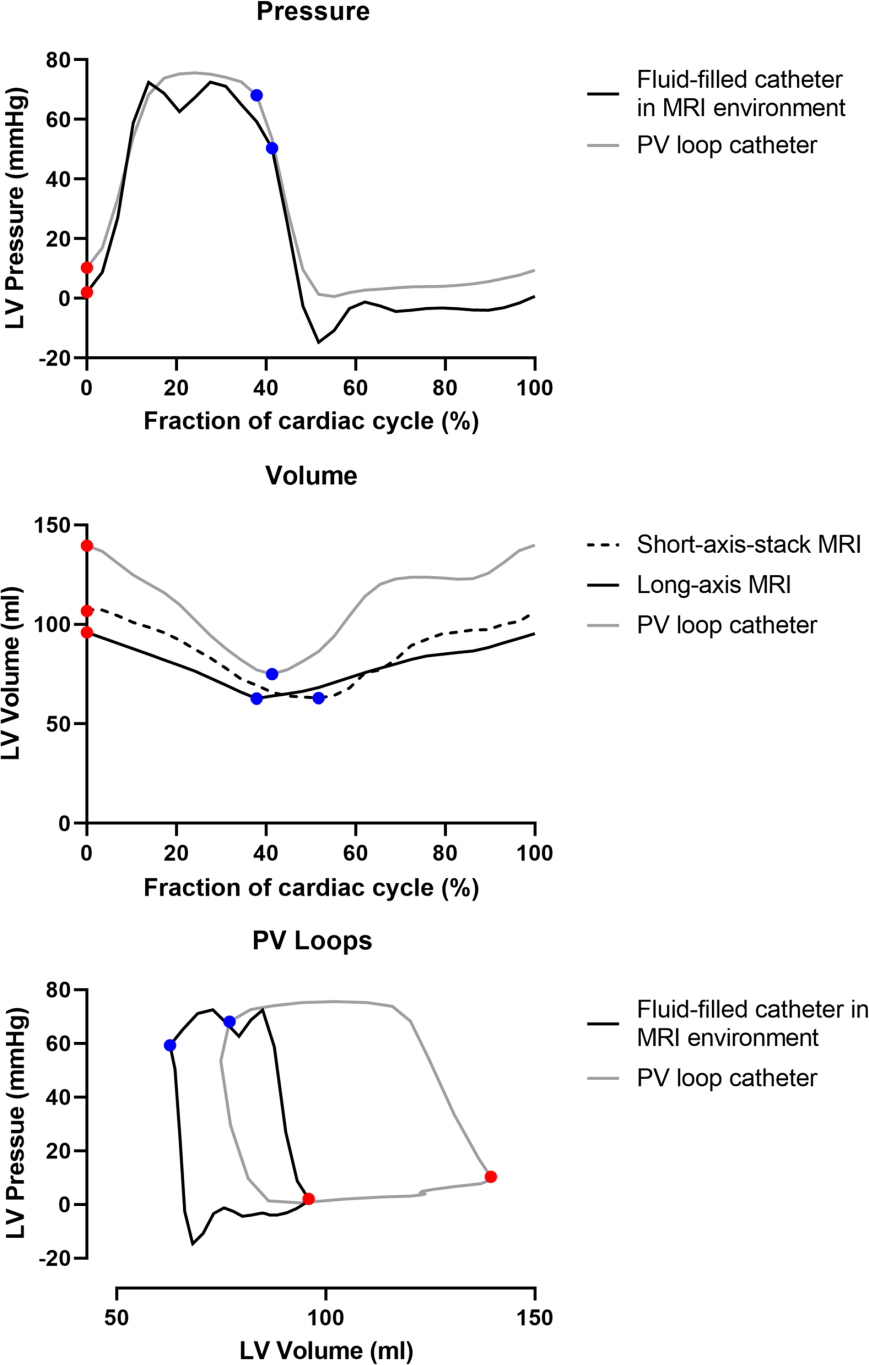
Example of LV pressures and volumes in a naïve animal, measured in the CMR environment (black) and with a conductance PV loop conductance catheter (gray). Corresponding illustrations of end-diastole and end-systole time points are shown in red and blue, respectively. **A** LV pressure measured with a fluid-filled catheter in the CMR environment and with a conductance PV loop catheter. Damping was visible in systole and diastole in the fluid-filled pressure measurement. **B** LV volume measured with short-axis-stack CMR (dashed black line), long-axis real-time CMR, and a PV loop catheter. Volumes measured with real-time CMR were comparable to reference standard short-axis-stack over the entire cardiac cycle, while the PV loop catheter measured larger volumes despite being calibrated using short-axis CMR volumes. **C** PV loops derived from combined pressure and volumes measurements in the CMR environment using real-time long-axis volumes, and with a PV loop catheter

LV volumes derived from real-time long-axis imaging and centerline rotation across all animal models had an excellent agreement with short-axis-stack imaging at both end-diastole (ICC = 0.97, R = 0.98, p < 0.001, bias 7.6 ± 9.2% or 8.2 ± 14.5 ml) and end-systole (ICC = 0.99, R = 0.99, p < 0.001, bias − 4.2 ± 8.6% or − 2.8 ± 6.5 ml). Figure 3 provides correlation plots and Bland–Altman comparison of the two measurement methods and Fig. 4B provides an example comparison of the cardiac volume over time in a naïve animal. Volumetric biases across the different animal models at end-diastole and end-systole were respectively 8.4 ± 6.5 ml and − 2.9 ± 5.3 ml in the naïve animals, 9.8 ± 10.0 ml and − 3.3 ± 7.9 ml in the aortic banding animals, and 0.7 ± 8.4 ml and 4.7 ± 34.4 ml in the cardiomyopathy animals. Cardiomyopathy animals were larger (83 ± 9 kg) than naïve (50 ± 6 kg) and aortic banding animals (46 ± 7 kg), and therefore had larger ventricles, stroke volume, and cardiac output compared to the other animal models. The low LV ejection fraction (LVEF) in the cardiomyopathy animals was expected (27 ± 4.5%). However, both naïve and aortic banding model pigs used in this study had mildly reduced LVEF at baseline (39 ± 12% and 36 ± 10%, respectively). This could be due to an increased sensitivity to amiodarone, which we use as a routine pre-medication in the catheter laboratory, in this cohort.

### Dynamic pressure–volume loops

**Fig. 5:**
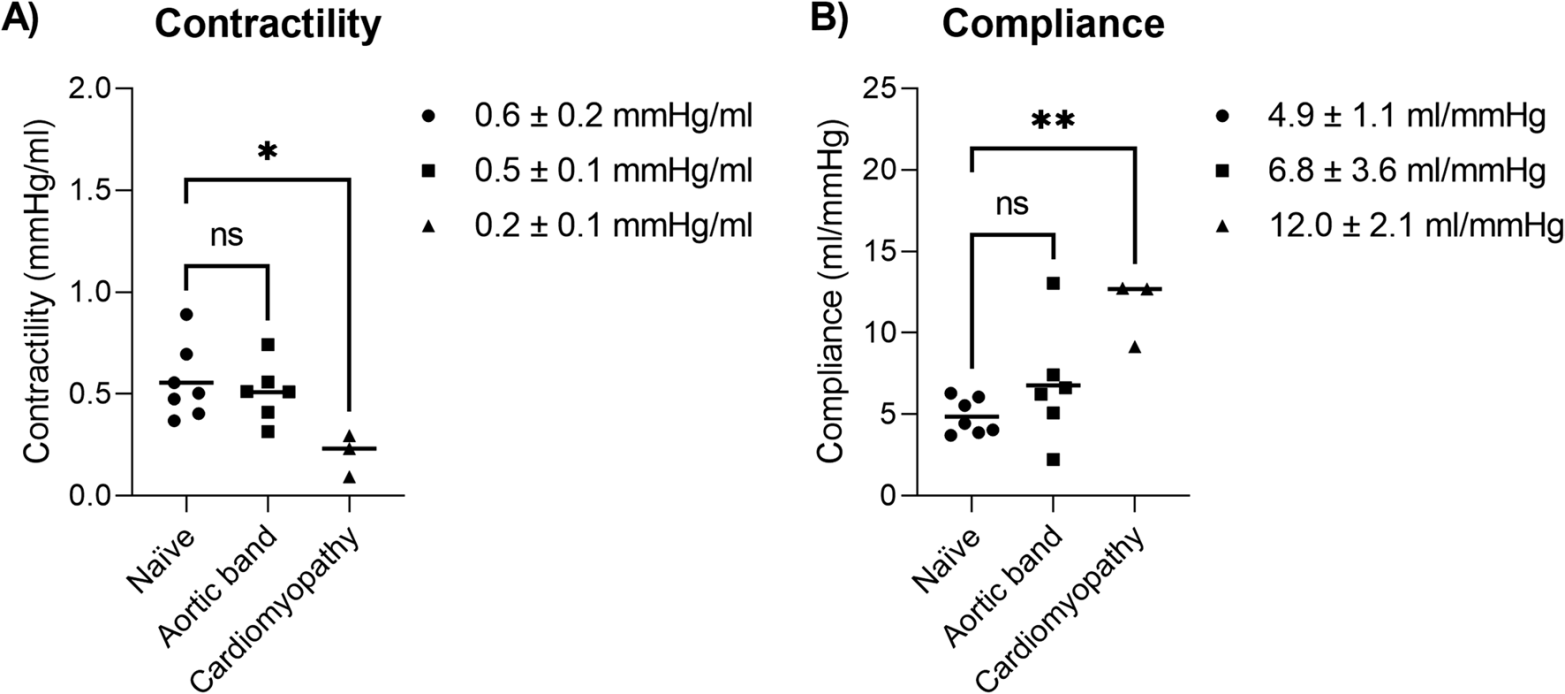
Comparison of **A** contractility and **B** compliance in the naïve, 30 days after aortic banding, and ischemic cardiomyopathy animal models. Quantitative values were derived from measurements in the CMR environment and are reported as mean ± standard deviation. Animal models were unpaired. *ns* non-significant; *p < 0.05; **p < 0.01

Quantified contractility and compliance are shown in Fig. 5. We detected differences in contractility and compliance between naïve and cardiomyopathy models, but not between naïve and banded models.

The cardiomyopathy animal model developed a dilated ventricle (short-axis EDV 277 ± 36 ml, ejection fraction 27.0 ± 4.5%), with lower contractility (0.2 ± 0.1 mmHg/ml vs 0.6 ± 0.2 mmHg/ml, p < 0.05) and increased compliance (12.0 ± 2.1 ml/mmHg vs 4.9 ± 1.1 ml/mmHg, p < 0.01) compared to naïve animals. We measured no difference between aortic banding model and naïve animals in contractility (0.5 ± 0.1 mmHg/ml vs 0.6 ± 0.2 mmHg/ml, p = 0.82) and compliance (6.8 ± 3.6 ml/mmHg vs 4.9 ± 1.1 ml/mmHg, p = 0.32), which is explained by the 10 ± 6 mmHg pressure gradient across the aortic band, that was considered modest and not clinically significant [25].

### Comparison with conductance catheter pressure–volume loops

An example of single-beat LV pressures, volumes, and PV loops measured in the CMR environment and with a conductance catheter is shown in Fig. 4, measured before IVC occlusion in a naïve animal. The example highlights strengths and shortcomings of both the CMR and catheter methods. The conductance catheter pressure measurement was steadier than the fluid-filled catheter pressure, as expected. Fluid-filled catheters are inherent sensitive to damping, which in this example was apparent both peak systole and early diastole, and is likely ascribed to an air bubble in the fluid column. Long-axis real-time CMR volume measurements were on the other hand comparable to the reference standard short-axis-stack volumes, whereas the conductance PV loop catheter in this example measured higher volumes throughout out the entire cardiac cycle, despite being calibrated with short-axis cine volumes. Differences in pressure and volume measurements were also perceptible in the combined PV loop representation, resulting in different positioning of end-systolic and end-diastolic detections in the PV plane. These different positions will impact the derivation of the ESPVR and EDPVR lines, and in turn, the quantification of contractility and compliance.

**Fig. 6:**
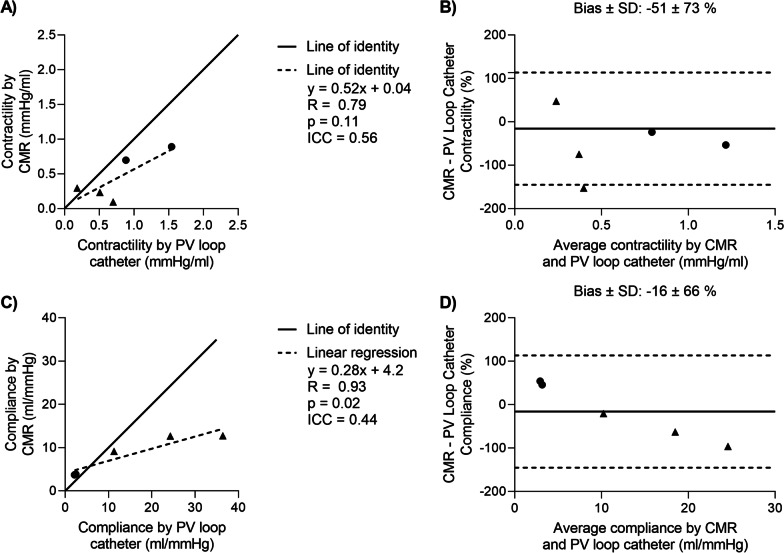
Comparison of contractility and compliance measured in the CMR environment and with a conductance PV loop catheter. Naïve animals are shown as circles, and ischemic cardiomyopathy animal models as triangles. **A** Scatter plot of contractility. **B** Bland–Altman plot of contractility. Bias and limits of agreement are shown as solid and dashed lines, respectively. **C** Scatter plot of compliance. **D** Bland–Altman plot of compliance. *ICC* intraclass correlation coefficient, *SD* standard deviation

The impact on quantitative parameters is illustrated in Fig. 6, showing correlation plots and Bland–Altman comparison of contractility and compliance derived from measurements by CMR and a conductance PV loop catheter. There was a weak to moderate agreement and large bias for both quantitative parameters (contractility ICC = 0.56, bias − 51 ± 73%, compliance ICC = 0.44, bias − 16 ± 66%), but no statistically significant differences were found in contractility (0.44 ± 0.33 mmHg/ml vs 0.76 ± 0.51 mmHg/ml, p = 0.31) or compliance (8.4 ± 4.5 ml/mmHg vs 15.0 ± 15.0 ml/mmHg, p = 0.35) quantified from dynamic PV loop measurements in the CMR environment vs with a conductance PV loop catheter. Real-time long-axis volumetric measurements and pressure measurements from the CMR environment are compared to conductance PV loop catheter measurements in Table 2, disclosing differences in ESV, ejection fraction, cardiac output, and peak LV pressure between the methods. Volumes measured at baseline with a conductance catheter and short-axis-stack volumes did not differ significantly at end-diastole (EDV: 220 ± 94 ml vs 205 ± 102 ml, p = 0.08, bias 11 ± 13% or 15 ± 15 ml), or end-systole (ESV: 143 ± 73 ml vs 145 ± 82 ml, p = 0.84, bias 4 ± 13% or 2 ± 16 ml).

**Table 2:** Quantitative measurements using real-time long-axis images and fluid-filled catheters in the CMR environment vs the conductance PV loop catheter

	CMR environment	Conductance PV loop catheter	t-test p-value
Heart rate (bpm)	79 ± 11	82 ± 11	ns, p = 0.60
EDV (ml)	198 ± 107	220 ± 94	ns, p = 0.21
ESV (ml)	144 ± 83	143 ± 73	ns, p = 0.84
SV (ml)	54 ± 24	78 ± 22	*, p = 0.04
EF (%)	29 ± 5	38 ± 9	*, p = 0.02
CO (l/min)	4.1 ± 1.5	6.3 ± 1.5	*, p = 0.02
LV EDP (mmHg)	8.8 ± 5.0	10.4 ± 2.1	ns, p = 0.42
LV ESP (mmHg)	70.6 ± 7.5	62.8 ± 5.6	ns, p = 0.13
Peak LV pressure (mmHg)	81.8 ± 5.1	69.3 ± 4.5	*, p = 0.02

Parameters were derived from the from the first recorded heartbeat prior to inferior vena cava occlusion in the dynamic pressure–volume loop experiments. CMR measured HR, EDV, ESV, SV, and CO are reported from the long-axis cine images. Continuous variables are reported as mean ± SD. *CMR* cardiovascular magnetic resonance, *EDV* end-diastolic volume, *ESV* end-systolic volume, *SV* stroke volume, *EF* ejection fraction, *CO* cardiac output, *LV* left ventricular, *EDP* end-diastolic pressure, *ESP* end-systolic pressure. *ns* non-significant; *p < 0.05; **p < 0.01

## Discussion

This study presents a method to derive dynamic PV loops during a preload alteration challenge by pairing simultaneously measured LV pressures via invasive catheterization with CMR-specific signal conditioning, and LV volume via real-time cardiac CMR in the long-axis view without the need for volumetric calibration. The PV loops were used to derive functional parameters of cardiac contractility and compliance, which were evaluated in three animal models. Long-axis derived volumes were validated with short-axis-stack cardiac images. The agreement of contractility and compliance derived from PV loops measured in the CMR environment and with a conductance PV loop catheter was weak to moderate.

### Dynamic pressure–volume loop analysis

In this study, we approximated the ESPVR and EDPVR to linear relationships and derived contractility and compliance from their respective slopes. The ESPVR is approximately linear within the physiological range of end-systolic pressures and volumes, whereas the EDPVR is exponential [26]. A linear approximation of the EDPVR has however been shown to suffice as a quantitative metric of compliance characterizing the overall diastolic function [27], as the slope of and exponential varies greatly depending on loading conditions.

Our method was able to characterize both a decreased contractility and increased compliance in animals with cardiomyopathy compared to naïve animals. These results are consistent with changes seen in dilated cardiomyopathy in humans [26]. Conversely, a decrease in compliance due to an increased afterload was expected but not observed in the animals with aortic banding. This negative result is explained by the clinically non-significant pressure gradient that developed across the banding. Future studies using another animal model with a significantly increased afterload, as well as evaluation in other diseases, are warranted to further evaluate the efficacy of the proposed method.

### Pressure measurements

Intracardiac pressures can only be measured invasively, often using either a fluid-filled catheter transducer or a catheter equipped with a solid-state pressure sensor. Solid-state pressure sensors are positioned on the catheter and therefore measure pressure at the site of interest, a technique typically used in conductance PV loop catheters. Unfortunately, these catheters are not CMR-compatible. Fluid-filled catheters offer the advantage of being CMR compatible, but measure pressures that have been translated through the fluid column to a transducer located outside of the body. Fluid-filled catheters are therefore inherently susceptible to damping and latency, specifically at peak systole and early diastole, especially if there is an air bubble in the catheter [28]. In this study, we observed damping in some experiments performed with fluid-filled catheters in the CMR environment, but not in the conductance catheter experiments. We also measured higher peak systolic LV pressures with the fluid-filled catheter compared to conductance catheters, which may be explained by underdamping in the fluid-filled pressure transducer [28]. However, this damping did not nominally impact pressures at end-systole and end-diastole, meaning that the pressures at the sampled points used to derive ESPVR and EDPVR, and in turn, contractility and compliance, should not be affected by this limitation. Other hemodynamic parameters that may be of interest might be impacted, for example stroke work which is measured as the area inside the PV loop. A catheter with a solid-state pressure sensor would thus theoretically be preferable to the fluid-filled catheter for this application, if CMR compatible.

Another challenge of the CMR environment is the RF-induced interference on physiological signal recordings. In this study, this was evaded by filtering out CMR-induced noise using the custom open source PRiME system [17]. The PRiME system was also used to ensure that only physiological signals recorded simultaneously to the real-time CMR sequence acquisition were sampled for the PV signal pairing. The simultaneous and synchronized ECG measurements also allowed detection of end-diastole from the straightforwardly detected peak R-wave rather than from the LV pressure signal.

### Volume measurements

Short-axis-stack cine CMR is considered reference standard for measuring intracardiac volumes and is widely used in the clinical setting [16], but typically requires several minutes of image acquisition to produce a cine image of one heartbeat. In order to capture the transient LV volumes during IVC occlusion, we opted to measure volume from a real-time long-axis 4-chamber cine by performing center line rotation on 2D LV segmentations. These long-axis derived volumes were comparable to short-axis-stack volumes without a need of volumetric calibration, with a clinically acceptable bias of < 10 ml which held across the three animal models [29]. Center line rotation does however approximate the LV to a perfect circular shape around the rotated center line axis. This approximation was sufficient in our study, but may introduce bias in subjects with a more irregularly shaped ventricular cavities or with regional wall motion abnormalities not depicted in the long-axis view. Conductance PV loop catheters assume a cylindrical LV geometry and provide relative volume estimates using measures of the time-varying electrical conductance induced by electrodes on the catheter, using an approximation of the myocardial parallel conductance derived from the hypertonic saline calibration procedure [30, 31]. A secondary calibration is then required for absolute volume estimations, which in this study was performed using ventricular volumes measured by short-axis CMR. We found that these calibrated conductance PV loop catheter volumes were not comparable to end-diastolic short-axis-stack CMR with bias > 10 ml, confirming previous findings by Jacoby et al. [8]. The volumetric discrepancy impacted computation of both contractility and compliance, resulting in a weak to moderate agreement between conductance catheter and CMR-derived measurements of these parameters. Quantitative values of contractility and compliance derived from dynamic PV loops measured in the CMR environment and conductance catheter are therefore not directly comparable. Given the superiority of volumetric measurements in CMR, this technique may provide the more accurate value.

The temporal resolution of the conductance catheters is significantly higher than CMR (4 ms vs 76 ms in this study). Conductance catheters are therefore better at characterizing the short isovolumic phases of the cardiac cycle (IVCT ~ 50 ms, IVRT ~ 80 ms) [32]. Witschey et al. previously found that an CMR temporal resolution of < 100 ms is sufficient to derive reliable PV loops, and suggested a real-time sequence implementation with 95.2 ms temporal resolution [13]. The proposed method in this study provides a real-time temporal resolution of 76 ms. Nevertheless, a sequence with higher temporal resolution, for example using spiral or radial sampling [33, 34], would be desirable to better characterize the volumetric variations over the cardiac cycle, especially at higher heart rates where 76 ms would only capture a few timeframes per heartbeat. In addition to offering reliable volumetric measurements, other advantages related to CMR-guided PV loops include the lack of ionizing radiation compared to X-ray fluoroscopy which is used for conductance PV loop catheterization, and that the procedure can be combined with a comprehensive CMR examination for evaluation of for example tissue characterization and other volumetric assessments.

### Low field strength CMR

The contemporary 0.55 T CMR system used in this study is well suited for CMR-guided interventional procedures due to the reduced RF-induced heating of metallic devices, which is quadratically related to field strength [22]. Device heating was, however, not measured in this study as we used a polymer based catheter without metallic-braiding. Cardiac catheterizations under CMR guidance have also been performed at 1.5 T [10, 20, 35], and we anticipate that the proposed dynamic PV loop recording would translate to higher field strengths.

### Limitations

This study had some notable limitations, including a relatively small sample size of 16 pigs. Additionally, the different sampling frequencies of the measured signals warranted the need to down-sample pressure and up-sample volume signals. The lower temporal resolution in the CMR measurements compared to the pressure measurement might impact the short isovolumic phases of the cardiac cycle in the derived PV loop representations. Another limitation for both approaches we used, is that the approximation of the ESPVR to a load independent linear relationship assumes an unchanged myocardial inotropy [36]. The physiological response to a preload alteration challenge includes the activation of the sympathetic autonomic nervous system, leading to a change in inotropic conditions. To avoid fitting the ESPVR to end-systolic points at altered inotropic states, we limited the included number of heartbeats after IVC occlusion to ten, while also monitoring for a significant beat-to-beat increase in heart rate which in this study was low (0.77%). Including fewer heartbeats would result in a poor linear fit. Hence, when selecting the number of points used for fitting of the ESPVR line, there was a compromise between including enough data to derive a reliable linear fit while avoiding including too many points at an altered inotropic state. Furthermore, volumetric estimations from long-axis images may be adversely affected by asymmetric ventricles, regional wall-motion abnormalities, and through-plane motion in the case of inconsistent breath holds. Further evaluation of this volumetric estimation is warranted in larger numbers of subjects with ischemic cardiomyopathy. Future work could also explore a cine or a real-time 3D cine sequence.

## Conclusions

Dynamic PV loops can be recorded in the CMR environment. Preload reduction by balloon-IVC occlusion, simultaneous measurement of LV pressure from a fluid-filled catheter and ventricular volume from real-time CMR enable derivation of dynamic PV loops from which contractility and compliance are quantified. Real-time CMR PV loops provided more reliable volumetric measurements compared to conductance PV loop catheters calibrated with hypertonic saline and short-axis CMR volumes. CMR PV loops are a promising alternative to conductance catheter measurements and may provide a more accurate assessment of myocardial performance.

## Supplementary Information

**Additional file 1: Video S1**. Left Panel: Real-time 4-chamber long-axis image capturing a dynamic preload alteration by an abrupt inferior vena cava (IVC) occlusion via balloon inflation. The white arrow highlights the balloon, which after inflation results in a reduced left ventricular (LV) volume. Right Panel: Example of measured LV pressure over the course of 25 s. The arrow indicates the approximate time of the balloon inflation for IVC occlusion, after which the LV volume starts decreasing.

## Data Availability

Source code for signal processing is available as open source (https://github.com/NHLBI-MR/dynamic_pv_loops). The datasets used during the current study are available from the corresponding author on reasonable request.

## Abbreviations

CMR: Cardiovascular magnetic resonance
ECG: Electrocardiogram
EDPVR: End-diastolic pressure–volume relationship
EDV: End-diastolic volume
ESPVR: End-systolic pressure–volume relationship
ESV: End-systolic volume
ICC: Intraclass correlation coefficient
IVC: Inferior vena cava
IVRT: Isovolumic relaxation time
LV: Left ventricle/left ventricular
LVEF: Left ventricular ejection fraction
PRiME: Physiological recording in the CMR environment
PV: Pressure–volume
RA: Right atrium/right atrial
RF: Radio frequency
